# Floral Reversion in *Arabidopsis suecica* Is Correlated with the Onset of Flowering and Meristem Transitioning

**DOI:** 10.1371/journal.pone.0127897

**Published:** 2015-05-26

**Authors:** Amelia Asbe, Starr C. Matsushita, Spencer Gordon, H. E. Kirkpatrick, Andreas Madlung

**Affiliations:** Department of Biology, University of Puget Sound, Tacoma, Washington, United States of America; McGill University, CANADA

## Abstract

Angiosperm flowers are usually determinate structures that may produce seeds. In some species, flowers can revert from committed flower development back to an earlier developmental phase in a process called floral reversion. The allopolyploid *Arabidopsis suecica* displays photoperiod-dependent floral reversion in a subset of its flowers, yet little is known about the environmental conditions enhancing this phenotype, or the morphological processes leading to reversion. We have used light and electron microscopy to further describe this phenomenon. Additionally, we have further studied the phenology of flowering and floral reversion in *A*. *suecica*. In this study we confirm and expand upon our previous findings that floral reversion in the allopolyploid *A*. *suecica* is photoperiod-dependent, and show that its frequency is correlated with the timing for the onset of flowering. Our results also suggest that floral reversion in *A*. *suecica* displays natural variation in its penetrance between geographic populations of *A*. *suecica*.

## Introduction

Patterning of the plant body in angiosperms is dependent on the development of groups of stem cells that are kept in an undifferentiated state, called the meristem. As long as the plant develops vegetatively, the shoot apical meristem continually adds leaves, stems, and axillary buds to the above-ground part of the plant [1]. Genetic and environmental cues trigger changes in the meristem as the plant transitions from juvenile to reproductive phase. These changes cause a developmental switch from generating vegetative tissues from the vegetative meristem (VM) to producing an inflorescence meristem (IM), which gives rise to the inflorescence. As the inflorescence continues to grow it sets aside cells that form the floral meristems (FM), which ultimately produce the flowers [2]. Flowers are usually determinate structures of the angiosperm body plan that can result in the production of seeds to complete the plant’s life cycle. Flower formation and seed production can also set in motion the natural process of senescence, particularly in annual plants [3]. In perennial plants, flowering and vegetative growth can alternate between seasons over many years. Perennial plants often set aside vegetative meristems that do not undergo a change from juvenile to adult phase, allowing the plant to resume growth after flowering and restricting senescence locally within the plant body [4,5].

In some species, flowers can revert from committed flower development back to an earlier developmental phase. Such a reversal is called floral reversion [6], and can occur in the families Myrtaceae, Bromeliaceae, Hyacinthaceae and Liliaceae [7,8]. In some species flower reversion is induced by a change in environmental factors, such as temperature, light, or humidity. If such changes occur at a time when the meristem is not yet fully committed to flowering the removal of cues normally leading to flower induction can lead to reversion [9,10], potentially avoiding senescence at a time when flowering would not result in successful seed production.

Although the molecular foundation of flower development in model plants, such as *Arabidopsis thaliana*, has been intensely studied, the molecular pathways leading to meristem commitment are still incompletely understood. One reason for this lies in the fact that the major model species, such as *A*. *thaliana*, snapdragon, maize, or rice do not normally undergo flower reversion. Meristem commitment in *A*. *thaliana* occurs only in inductive conditions, which are perceived and integrated via multiple signal transduction pathways that ultimately converge on just a few integrator proteins, many belonging to the MADS box family of transcription factors [11–14].

Phenotypes similar to floral reversion in wild-type accessions of *A*. *thaliana* are rare. *A*. *thaliana* ecotype Sy-0 displays some form of floral reversion due to a gain-of-function variant of the RNA-binding protein *ART/HUA2*, which is responsible for late flowering in this ecotype by strongly activating the floral repressor *FLOWERING LOCUS C* (*FLC*) via an unknown mechanism, and thereby repressing *AGAMOUS* (*AG*) [15,16]. This leads to floral reversion with phenotypes similar to those seen in some mutant alleles of *ag*, but is only exhibited in early flowers [17]. Another phenotype with some similarity to floral reversion was described for *A*. *thaliana* with a mutation in the *KNUCKLES* (*KNU*) gene, which is induced by *AG* [18,19]. An involvement of *AG* was also indicated in a recent report where the expression of a version of *TARGET OF EAT 3* (*TOE3*) with artificial resistance to miR172 was implicated in the repression of *AG* and a loss of floral determinancy [20].

Possibly the only species exhibiting natural floral reversion for which molecular mechanistic information is available is *Impatiens balsamina*. Both the physiology and genetics of floral reversion have been described in some detail in this species. Molecular analysis in *I*. *balsamina* showed that unlike in *A*. *thaliana*, the *LEAFY* (*LFY*) homolog in *I*. *balsamina* was expressed in the meristem throughout the vegetative and flowering phases, and was also expressed in reverting tissues. *IbTERMINAL FLOWER1* (*TFL1*) was not involved in maintaining the meristem in either a terminal or reverting state [21], and while *IbAG* was required for organ specification it was not sufficient in establishing determinancy [21].

We use *Arabidopsis suecica* to study floral reversion. *A*. *suecica* is an annual allopolyploid native to Scandinavia that arose from its progenitor species *A*. *thaliana* and *A*. *arenosa* between 12K and 300K years ago in a single hybridization event [22,23]. We previously reported that *A*. *suecica* naturally displays incompletely penetrant floral abnormalities in a subset of its flowers, some of which are reminiscent of homeotic mutations, while the majority of abnormal flowers appears to present a form of photoperiod-dependent floral reversion [24]. These reverting flowers can either fully or partially revert, where partial reversion only leads to a swelling of the carpel with more or less developed new inflorescences inside, but carpels in these partially reverting flowers never break open to release the new inflorescence [24]. In the same study we also showed that floral reversion was more likely to occur earlier in development when plants were grown in short day conditions, compared to long day conditions, where reversions appeared to occur later in development [24]. Additionally, we observed differences in the expression of some floral promoters and inflorescence maintenance genes when comparing reverting and non-reverting tissues, including *SHORT VEGETATIVE PHASE* (SVP), *APETALA-1* (*AP1*), *SUPPRESSOR OF CONSTANS1* (*SOC1*), and *AGAMOUS-LIKE 24* (*AGL-24*) [24].

Here we further characterize the phenotype showing that reversion in *A*. *suecica* likely originates from the replum, and present data indicting that day length, flower initiation, and propensity to revert are interconnected, suggesting that these processes could be controlled by similar pathways.

## Material and Methods

### Plant Material

*A*. *suecica* seeds from a variety of locations in Finland and Sweden (S1 Table) were a kind gift from C. Josefsson (Vancouver Island University). The original seed donor was T. Säll (Lund University). The ecotypes were grown in 20°C incubators (or 25°C where indicated) at the University of Puget Sound with light/dark photoperiods of 8/16, 12/12, 16/8, and 24/0 hours, respectively. The light intensity during the day was ~250 μM m^2^s^-1^. Seeds were planted in 10 cm diameter pots in soil (Sunshine Mix #4; Sungrow Horticulture, Vancouver, Canada) without stratification or vernalization. Onset of flowering was defined as the day from planting when the inflorescence had reached 1 cm in height or the first flower had opened, whichever occurred first. Mature plants were inspected for floral reversions and the floral position along the inflorescence of reversions was recorded numerically from the base to the apex. A flower was marked as reverting if the original flowering scar was located more than a centimeter from the final silique(s) [24], if there was more than one silique growing from a single flower, or if a silique contained additional flower and stem tissue in addition to seeds. Siliques were classified as either normal (including infertile) or reverting.

### Microscopy

Light microscopy was performed using a Leica stereo dissecting microscope and a Nikon digital camera. Scanning electron microscopy (SEM) was performed on a Hitachi S3400N variable pressure scanning electron microscope. Tissue for SEM was neither chemically fixed nor sputter coated. Photographs were edited or colored using Photoshop software. Due to the size of the carpel exceeding the zoom capacity of the camera, SEM images are composites of multiple sub-views of the same carpel.

### Statistical analysis of reversion rates and flowering time

Data were statistically analyzed using R software (versions 3.0.3 and 3.1.2, R Development Team, 2011). Data were tested for normality using qqPlot analysis [25]. Constant variance was assessed by visual inspection of the plotted residuals. For both flowering time and reversion rate, a 2-way ANOVA plus Tukey post-hoc test was used to analyze the differences between ecotypes and day-length treatment. One-way ANOVAs followed by Tukey post-hoc tests were employed to compare responses within ecotypes and within day-length treatments.

To determine statistical significance for the difference in the position of peak reversion in the four light treatments, we smoothed the curve and analyzed peak position as follows: we differentially weighted reversion at each position along the inflorescence by multiplying the floral position value (positions 1–50) by the rate of floral reversion at each position (N = 50 for each treatment), which we called the “weighted position.” We then calculated a sliding average of ten values around each position. For the first four positions (1–4) we substituted the preceding values for the averages with zeros. Likewise, for the last five positions (46–50) we substituted the missing values from the sliding average with zeros. Using the resulting smoothed values we determined the weighted peak position of reversion. For all individuals from all populations at a given light treatment the means of weighted positions were calculated and compared using ANOVA to determine if the means of position at each of the four peaks differed statistically significantly between the four light treatments. Normality of the data set was tested using qqPlot analysis, which suggested some departure from normality. Transformation or removal of zero values did not improve normality significantly. Since the central limit theorem for large data sets (here N = 50 per treatment) states that a small departure from normality does not affect the outcome significantly for large data sets, we proceeded with the ANOVA using the untransformed data. To determine if reversion rates in *A*. *suecica* were correlated with flowering time, we performed a Pearson correlation test.

## Results

### Floral reversion originates from replum tissue in A. suecica

**Fig 1:**
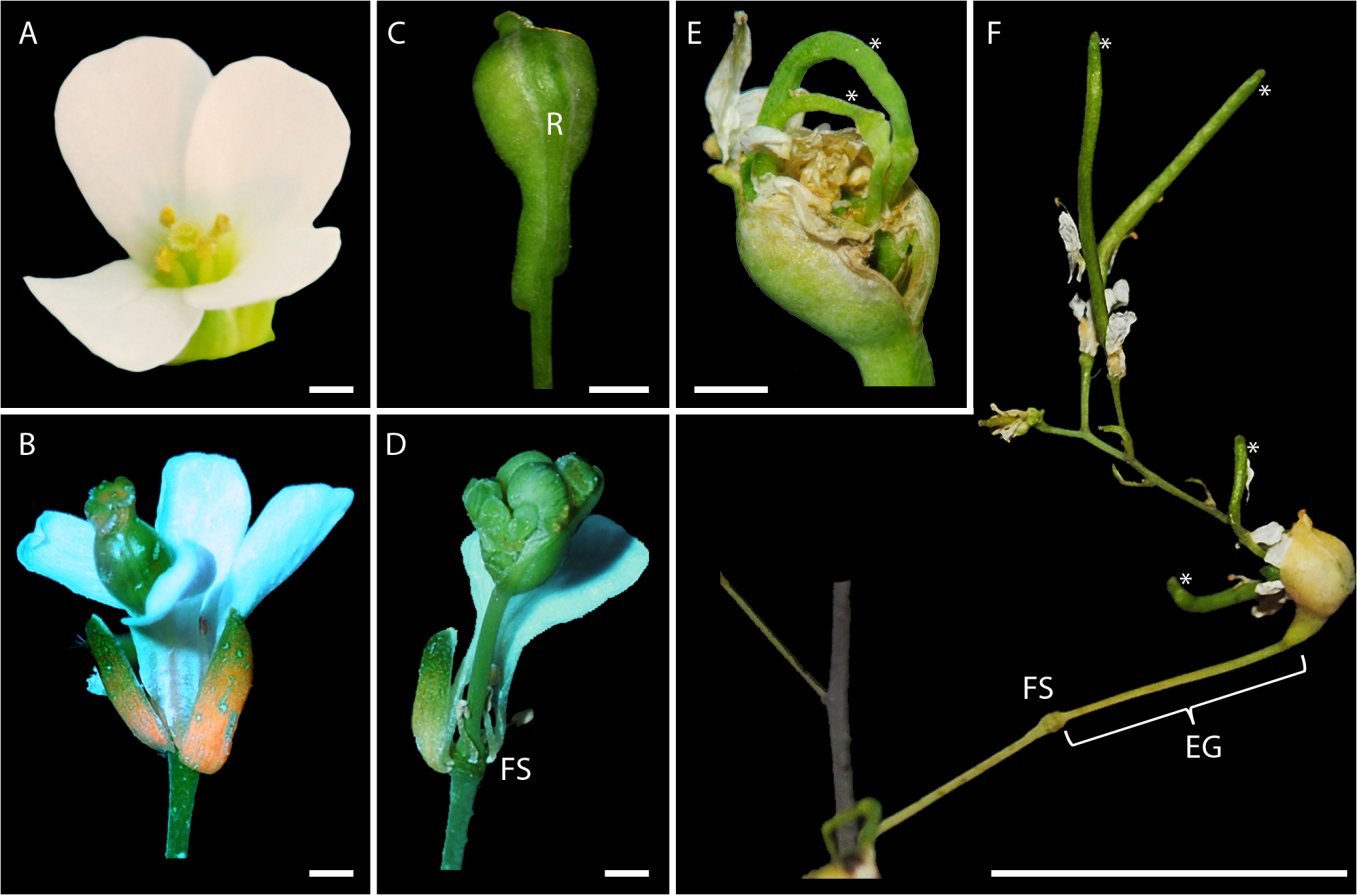
Floral reversion phenotype in *Arabidopsis suecica*. A: Normal flower. B: Reverting flower with a bulging carpel inside an otherwise normal flower. C: Isolated carpel from reverting flower. Note the unevenness of the size of the two valves at the basal end, and the stretched replum (R) between the valves. D: Reverting flower after petal fall. One petal remains marking the floral scar (FS). Note the burst-open carpel with approximately six additional flowers inside. E: Elongated siliques of the flowers inside a reverting carpel (asterisks). F: Reverting flower with the new inflorescence fully expanded. Four additional siliques are formed, three additional flowers are at petal fall stage. Note the elongated gynophore (EG) between the FS and the base of the reverting carpel. Scale bar = 1 mm (A-E) or 2 cm (F).

**Fig 2:**
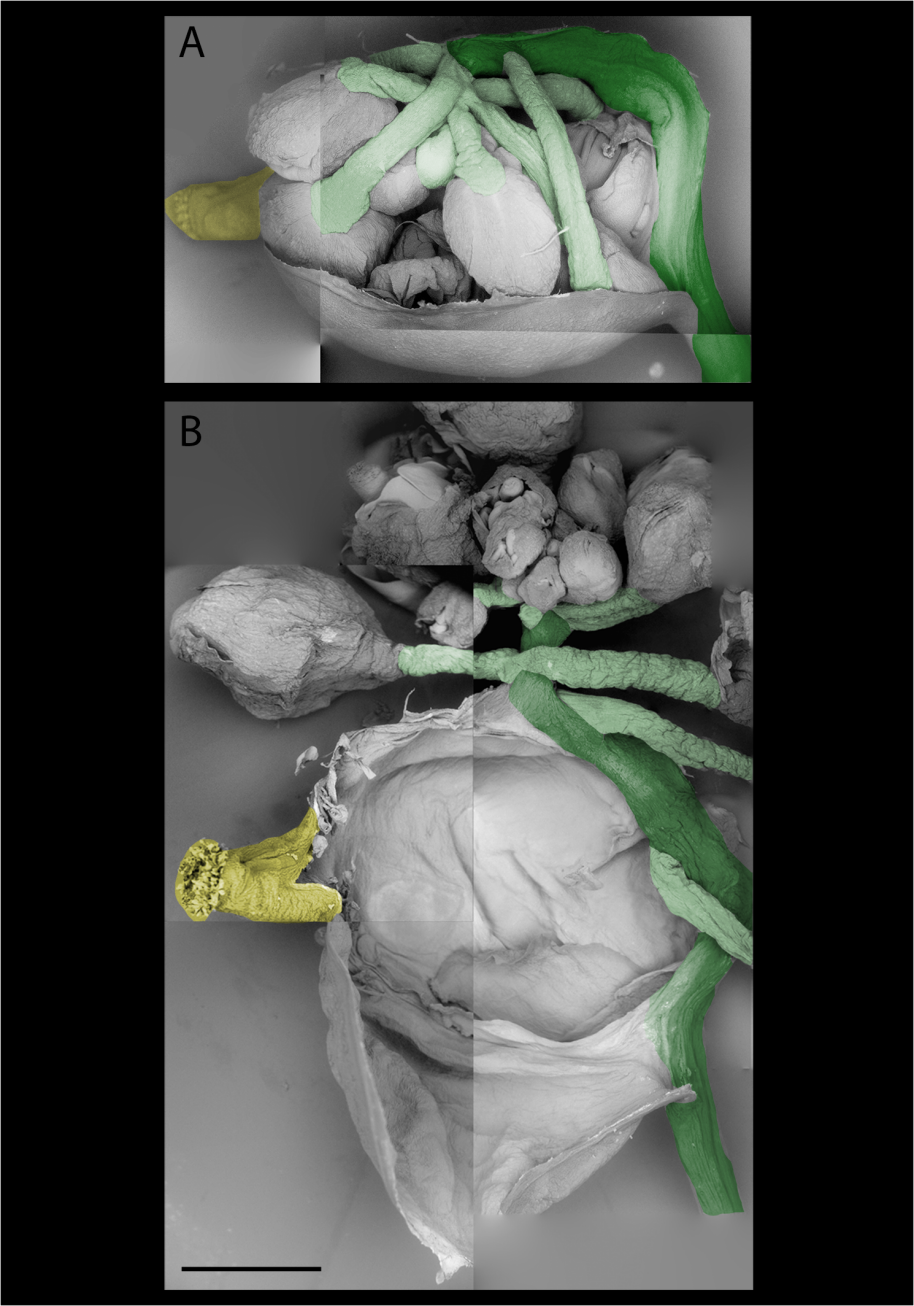
Floral reversion in *A*. *suecica* originates from the replum. A: The top valve of a reverting carpel was removed and the tissue imaged using environmental SEM. Multiple new floral stems (light green) originate out of the replum (dark green), which is connected to the gynophore (bottom right). The original stigma of the reverting flower is colored yellow for orientation. B: The same reverting carpel as in A. In this view the new inflorescence was unfolded and placed above its remaining carpel valve. Gynophore and replum are fused (dark green), and several new inflorescence stems branch off of the replum (light green). Stigma is colored in yellow as above. Colors are false colors. Size bar in B is the same as for A = 25 mm.

We have previously described floral reversion in *A*. *suecica* as an elongation of the gynophore, which forms the most basal part of the gynoecium [26], resuming growth after initial flower formation [24]. During complete floral reversion in *A*. *suecica* the carpel begins to swell, the gynophore elongates, the carpel eventually ruptures along the replum, which connects the silique valves [26], and new, fertile flowers, or sometimes already fertilized siliques emerge from the original carpel (Fig 1A–1E). New inflorescences emerging from reverting flowers can have multiple new, fully fertile flowers (Fig 1F). Incompletely reverting flowers stop the process of reverting at the swollen carpel stage (Fig 1C). To determine the origin of reverting tissue more closely, we used scanning electron microscopy (SEM). Removal of one of the two silique valves from the reverting tissue showed that the new inflorescence emerges from replum tissue, from which it subsequently branches out (Fig 2). New flower-bearing pedicels then branch off of the new peduncle (inflorescence stem) (Fig 2).

### Reverting flowers give rise to multiple new flowers with high fecundity

**Fig 3:**
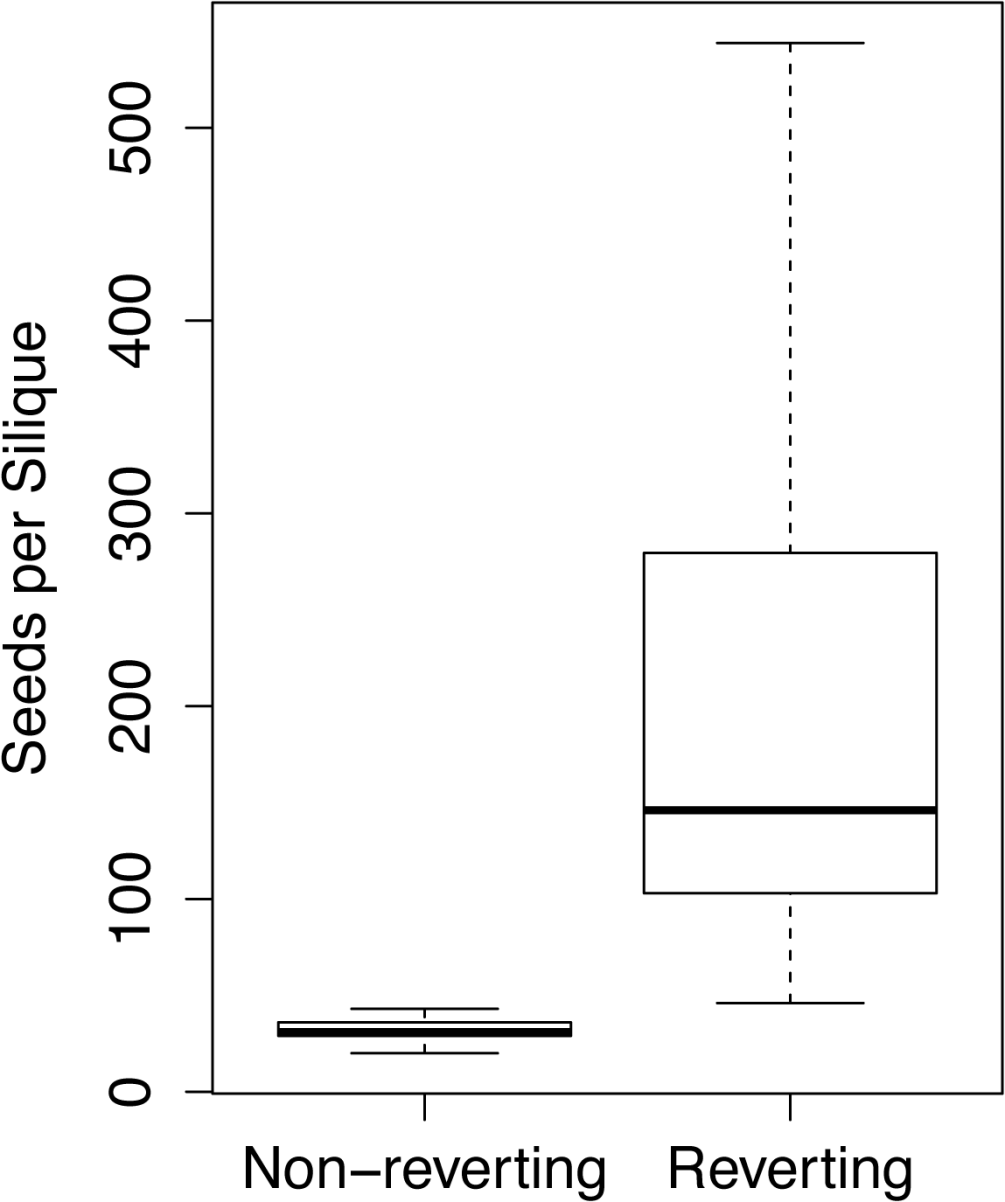
The total seed number is increased in reverting compared to non-reverting flowers. Seeds were harvested and counted from individual typical, non-reverting siliques (N = 27), and compared to the average of the sum of seeds from all flowers on new inflorescences (N = 21) erupting from reverting flowers. A two-sample Welch t-test, assuming unequal variances, indicated a statistically significant difference between the samples (t = 6.319; p < 0.001). Error bars reflect SE.

Since floral reversion multiplies the number of fertile flowers that emerge from each original flower position along the inflorescence stem we asked whether reverting flowers therefore were more fecund than single, determinate flowers. Comparing the sum of all seeds from flowers that fully reverted and gave rise to new inflorescences with single terminal flowers, we observed a clear, statistically significant difference between the two types suggesting that the abnormal developmental processes leading to reversion do not compromise flower fecundity, but rather increase it (Fig 3). Despite the increased fecundity of reverting flowers it is formally possible that high levels of reversion overall weaken the plant leading to a decrease in fecundity when assessing seed amount of the entire plant, as opposed to comparing a single flower to a new inflorescence arising from a reverting flower. To address this question we conducted two experiments. First, we compared plants that were grown at 20°C but kept at either 12h or 16h long days. Second, we grew plants at 25°C under 8h or 12h long days. We chose these conditions for two reasons: first, previous results showed that shorter days increase reversion frequency [24] therefore enhancing the difference between reverting and non-reverting plants. In addition, the high temperature condition allowed us to evaluate the possibility that the phenotype is due to a defect in *KNU*, a temperature-sensitive gene that affects flower determinancy [18]. Seed weight in both temperature conditions was unaffected by day length, regardless of the frequency of reversion on the whole plant. Growth at higher temperatures decreased the seed mass for both day length treatments equally compared to low temperatures (S1 Fig). These experiments suggested to us that overall fecundity was, by extension, unaffected by reversion rate, and *KNU* was not affecting the phenotype in any obvious way. Since reversion rates were not measured in these plants, no direct comparison between reversion and seed mass were made. Taken together, seed yield experiments suggest that individual reverting flowers increase the plant’s fecundity, while overall plant vigor is not affected by reversion.

### Frequency of floral reversion differs between populations of A. suecica

**Fig 4:**
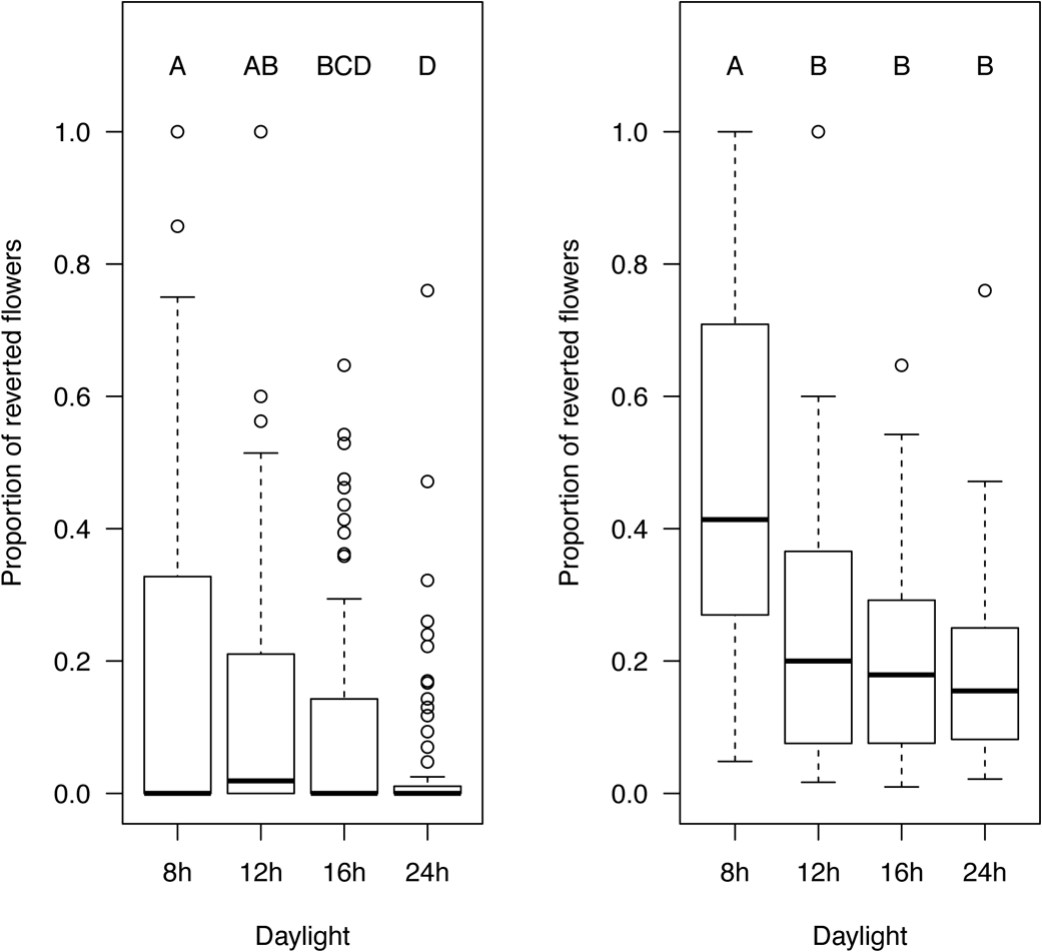
Floral reversion frequency increases in short days. Individuals from each population were grown in incubators (20°C; and day lengths set at 8h light/16h dark; 12h light/12h dark; 16h light/8h dark; 24h light) until senescence. Reverting flowers were counted along the inflorescence axis from the first (oldest) to the 80th flower position and the proportion of total reverting flowers was calculated for each condition. Here, data from all eleven populations were analyzed together. The plot on the left includes data from all plants from the study, the plot on the right excludes plants that exhibited no case of floral reversion. Statistically significant differences in reversion rates between treatments were determined using ANOVA and Tukey posthoc testing. Treatments not connected by a letter are statistically significantly different from each other (p< 0.05). Boxplots show data range, including median (bold line), the 25^th^, 75^th^, and 5^th^ or 95^th^ percentile of data distribution (lower and upper end of box, and lower and upper whiskers, respectively), plus outliers (circles). N = 148 (reverting), 190 (never reverting).

To address the question to what degree reversion frequency is genetically determined, we obtained geographically distinct populations (ecotypes) of *A*. *suecica* and asked whether or not there is natural variation in the rate of floral reversion among populations. Since *A*. *suecica* is an inbreeder different accessions are likely to be genetically homogeneous [27]. We first compared the frequency of this phenotype both between populations and within populations in response to day length. Several of the populations showed consistent and frequent floral reversions, while other populations displayed floral reversions only rarely or not at all (S2 Fig). Each of the populations originates from different latitudes within Sweden and Finland where they are naturally exposed to different day lengths during their floral development. To determine if day length affects reversion we analyzed our data in two ways: first, we pooled the data from all populations either including plants from our experimental populations even if they did not show any reversions (Fig 4, left panel), or including only those plants that showed at least one reverting flower and excluding plants with no reversions at all (Fig 4 right panel). Both types of analysis showed the same trend: frequency of reversion decreases with an increase in day length (Fig 4). We also compared the frequency of reversions within each individual population grown under different day lengths (S2 Fig; S1 Table). As expected, the data from the individual population analyses also show that overall longer days result in less frequent floral reversions, although statistical analysis only showed statistically significant changes for a subset of the populations (S2 Table).

Given that separate populations of *A*. *suecica* grow throughout Sweden and Finland, and are therefore exposed to different maximal day lengths in the summer depending on the latitude of their location we asked if the original location of collection correlated with their propensity to exhibit floral reversion but we found no statistically significant correlation between latitude of origin and frequency of reversion (data not shown). Taken together these data suggest that populations geographically isolated from each other can vary in reversion phenotype and that reversion rates generally decline with an increase in day length, but that the magnitude of the response is not genetically hard-wired by geographic origin.

### Peak position of floral reversion along the inflorescence axis is affected by day length

**Fig 5:**
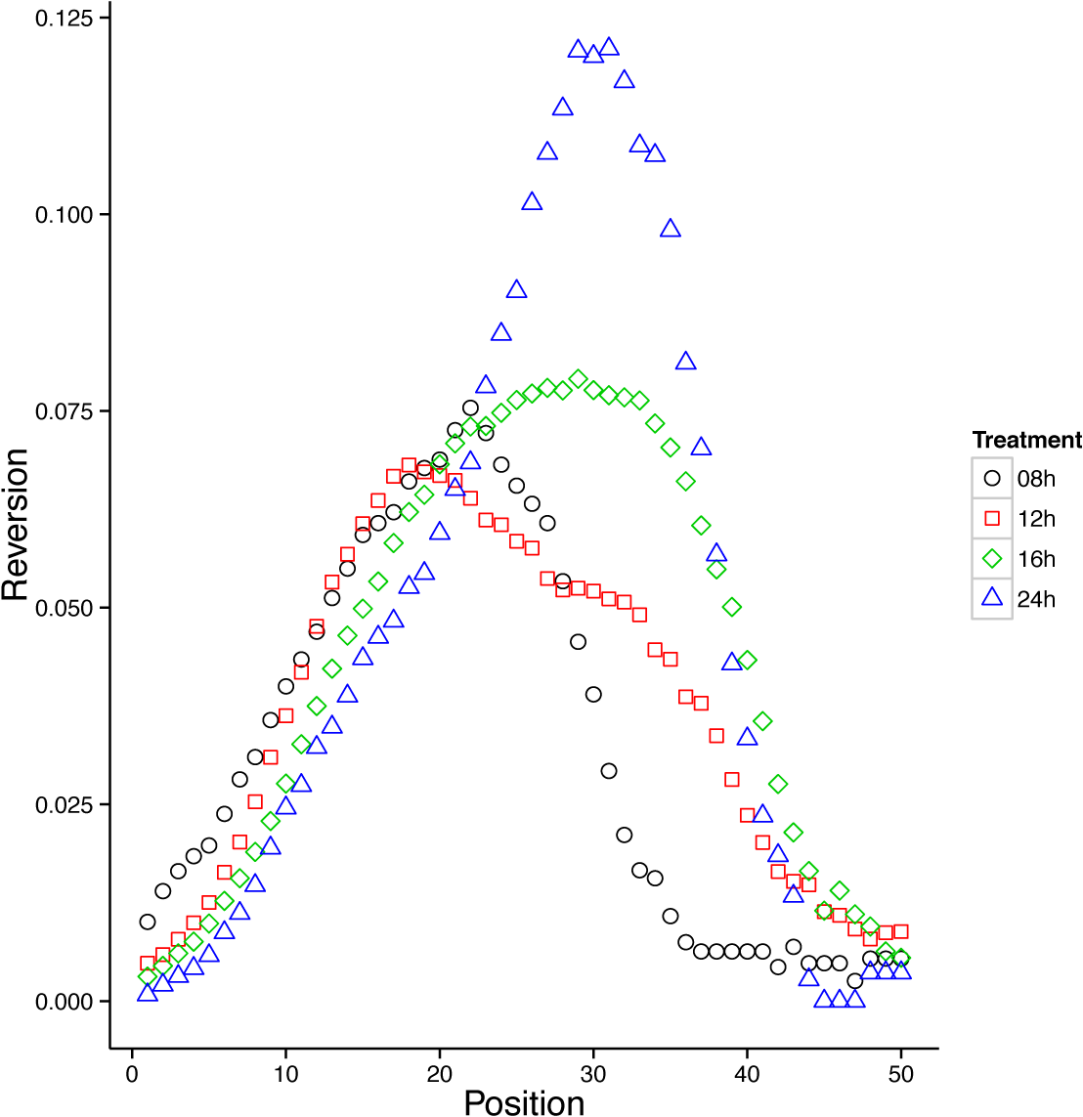
Floral reversion is more likely to occur earlier in development when days are short. To determine if reversion was equally likely for each position along the inflorescence from the oldest to the 50^th^ flower, “weighted reversion frequencies” (see Methods for details) were calculated for each day length condition and plotted. Statistical significance between the positions of the peaks was calculated using ANOVA and Tukey post hoc testing. Statistically significant differences in peak positions for reversions were found only for the two extreme values of 8h and 24h of light.

To further determine the effect of day length on the reversion phenotype we investigated where along the inflorescence axis reversion was the most frequent. To do so we plotted the “weighted reversion frequency” (see Methods for details) against the floral position along the inflorescence axis starting from the oldest flower closest to the base to the youngest flower closest to the apex (Fig 5). Peak reversion frequency along the inflorescence varied from position 18 (at 12h days) to position 31 (at 24h days). To analyze if this difference was statistically significant we used ANOVA and Tukey post-hoc testing to compare the means of the weighted peak positions (see Methods for details). While ANOVA suggested significance for the model (F = 2.751 on 3 and 196 df, p = 0.043), Tukey post-hoc testing only resulted in significant differences for the peaks between 8 and 24 hours (p = 0.046). Thus, the overall trend of this analysis suggests that positioning of reverting flowers along the inflorescence axis is significantly influenced by day length.

### Flowering time in A. suecica is affected by day length

**Fig 6:**
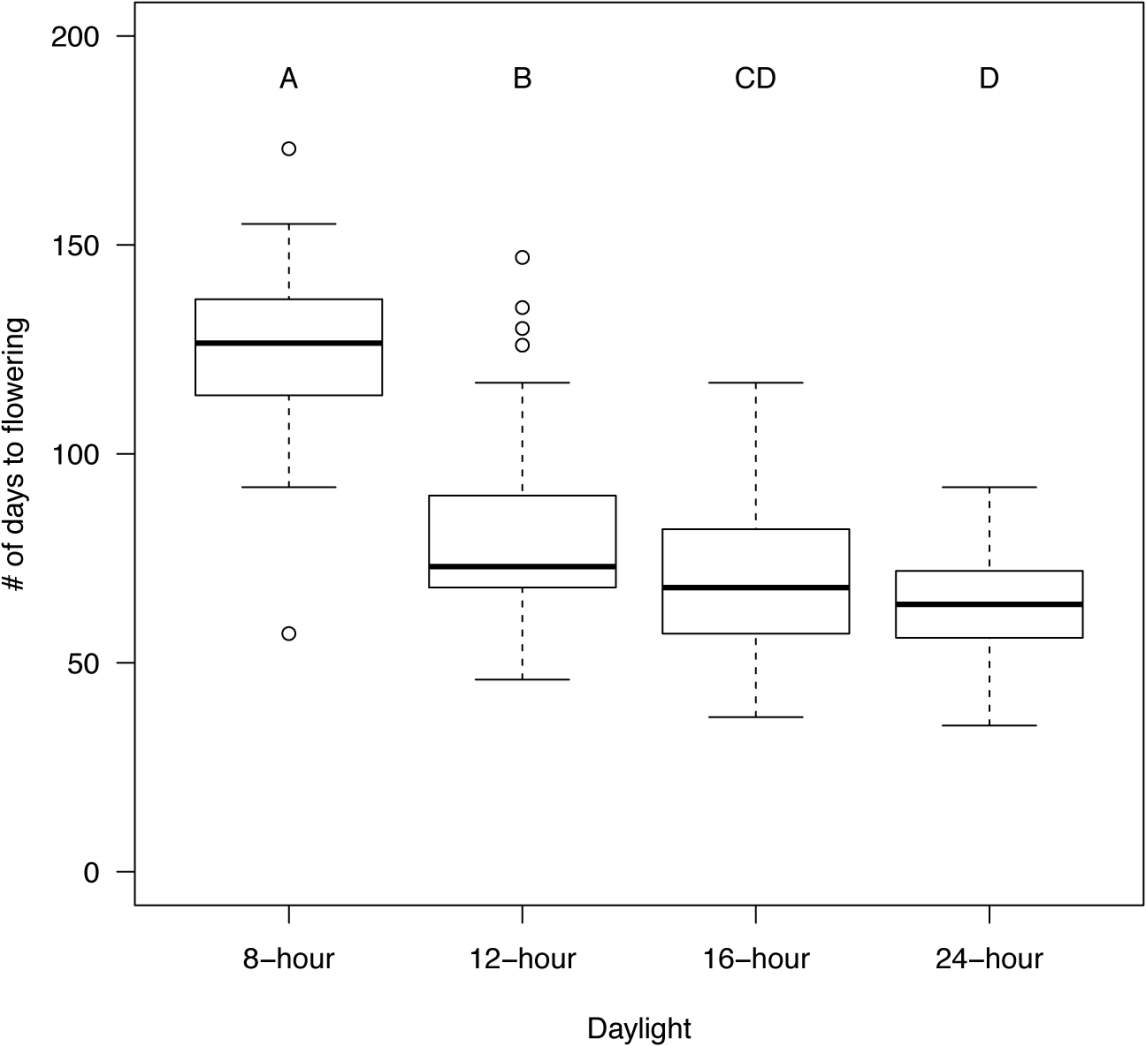
Flowering time is significantly increased in shorter days. Plants of eleven populations were grown in growth chambers at the indicated day lengths. The first day of flowering was defined as the day when the first flower opened or the inflorescence was 1 cm tall, whichever occurred first. ANOVA, followed by Tukey post hoc testing was performed for the combined data set of all populations of *A*. *suecica* (N = 270; data for all individual populations can be found in S3 Fig). Treatments not connected by the same letter are statistically significantly different. (For general boxplot description see Fig 4.)

Casual observation suggested that populations did not flower at the same time and that later flowering coincided with more reverting flowers. To validate this observation and to determine if flowering time in diverse populations of *A*. *suecica* varied, we measured the time from sowing to the opening of the first flower. We found that regardless of the geographic origin of the population *A*. *suecica’s* flowering time was significantly affected by day length. All eleven populations showed a decrease in flowering time from 8h photoperiod to 24h photoperiod with the greatest difference between 8h and 12h days and the smallest effect when day length was increased from 16h to 24h (Fig 6, S3 Table). Despite the increase in flowering time, 8h days were still sufficiently long to induce flowering in most individuals. We also noted significant differences in the flowering response between populations. The flowering time in the tested populations, grown in 8h days, varied between 97 and 157 days, and between 43 and 76 days for 24h days. Using ANOVA, we compared flowering times in two ways. First, we combined flowering times for all populations and analyzed the variation of the combined set by day length treatment (Fig 6). The second type of statistical analysis compared the flowering time response to day length treatment in each individual population (S3 Table). ANOVA and Tukey post-hoc analysis of the combined data set separated the four treatments into 3 statistically significantly different groups (Fig 6, Df: 3, 265, F = 112.8, p<2e-16). ANOVA for each individual population showed statistical significance for each analysis, and post-hoc testing resulted in variation in the number of statistically significantly different groups in these populations between 2 to 4 (S3 Table), suggesting that the response to variation in day lengths leads to variable magnitudes of the flowering time response depending on the population tested.

### Reversion frequency is positively correlated with flowering time in A. suecica

**Fig 7:**
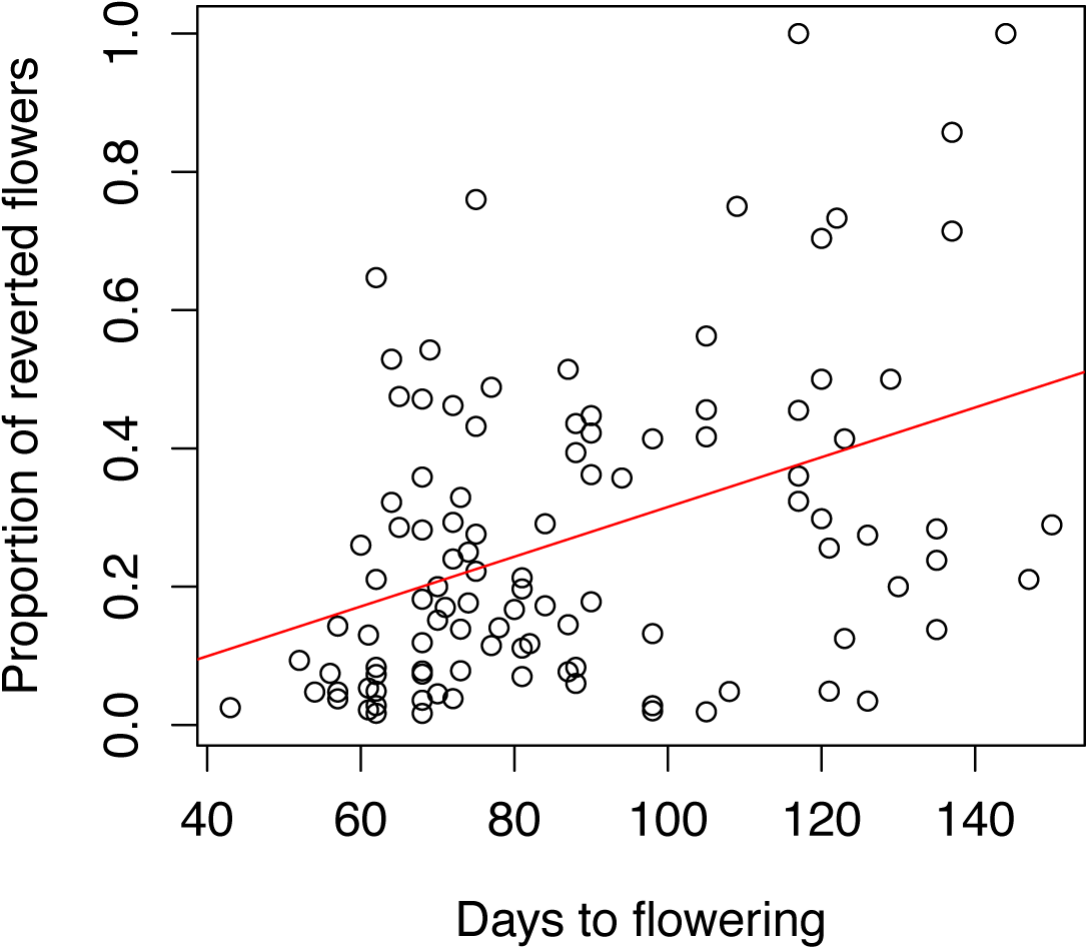
Floral reversion is positively correlated with flowering time. Flowering time and reversion data were plotted for each individual plant that showed any reversion, and the Pearson correlation coefficient was calculated (p< 0.0001, R^2^ = 0.169, N = 148).

Although the latitude of origin did not appear to effect reversion frequency or flowering time, it is possible that floral reversion frequency and flowering time are linked. To test this hypothesis we asked if flowering time and reversion frequency were correlated. We analyzed our data in two ways. First, arguing that plants that do not revert at all might obscure any relationship between the two parameters, we excluded all plants from the analysis that did not show any reversions (Fig 7). Pearson analysis resulted in a weak to moderate, albeit significant correlation suggesting a relationship between longer time to flowering and more frequent reversions (F = 21.35, df = 1,105, R^2^ = 0.169, p<0.0001). In a second analysis (S4 Fig) we included all plants, even those that did not revert. As expected, the strength of the correlation decreased but Pearson analysis still showed a significant correlation between flowering time and reversion frequency (F = 32.31, df = 1,266, R^2^ = 0.1083, p<0.0001). We cannot say if reversion rates are influenced directly by the plant’s age or developmental state at which flowering occurs, or indirectly by the day length dictating flowering time. Nonetheless, together these data show that additional time spent in the vegetative phase is correlated with an increased likelihood of floral reversion later in life.

## Discussion

We have been developing the allopolyploid *A*. *suecica* as a model system to better understand the developmental pathways leading to the orderly transition from an inflorescence meristem to a floral meristem. *A*. *suecica* displays a photoperiod-dependent, abnormal floral phenotype reminiscent of floral reversion [24]. We sought a) to more completely describe the floral reversion phenotype in *A*. *suecica*, and b) to better understand the processes that relate flower development and floral reversion to each other.

Using light and electron microscopy, we frequently found that reverting sectors emanated from the replum of the developing carpel (Figs 1 and 2), which itself extends out from the flower pedicel. The smooth transition of tissue types from pedicel, to replum, to new inflorescence stem in reverting flowers suggests that tissues in these floral structures retain the ability to initiate genetic programs of earlier developmental phases. A recent study suggested that inflorescence architecture in tomato is determined by a process organized by a suite of genes, and that delaying this meristem maturation process can lead to additional branching in inflorescences [28]. Applied to our findings, this might suggest that the structures we observed in floral tissues initiate new branching due to the delay in maturation, and thus result in a new inflorescence.

Reverting flowers reliably displayed an elongated gynophore (Fig 1F), regardless of whether the reversion phenotype was incomplete/partial (as in Fig 1C) or complete with a new inflorescence with fertile siliques (Fig 1E and 1F). An elongated gynophore is normally not seen in most species in the Brassicaceae, however, there are a few exceptions, where gynophore elongation is typical, such as in *Lunaria annua* [29]. Additionally, several mutations in *A*. *thaliana* have also been described that result in an elongated gynophore [26,30], although none of them result in phenotypes as drastic with respect to gynophore elongation as floral reversion does in *A*. *suecica*. Given these observations, it seems possible that floral reversion in *A*. *suecica* results in the partial use of an evolutionarily conserved pathway that leads to gynophore elongation in related congeneric species, but is typically suppressed in the genus Arabidopsis.

Reversion of individual flowers effectively alters the inflorescence architecture at each affected flower from an unbranched, indeterminate inflorescence with single flowers arranged along one axis (raceme) to a compound inflorescence with multiple smaller inflorescences branching off of the main axis (panicle). We asked if change in inflorescence morphology would lead to changes in fecundity of plants that display frequent reversions. While our data show that new inflorescences from individual reverting flowers produce about five times as many seeds as a typical flower (Fig 3), we did not see an overall increase in seed mass on a per-plant level in plants growing in conditions favorable to floral reversion compared to those favoring the formation of typical flowers (S2 Fig). Seeds from reverting flowers give rise to normal plants [24]. The fact that plants with any amount of reversions do not have overall increased seed mass might be in part explained by the observation that only a small subset of reverting flowers completes its development into fertile siliques and instead stops at an infertile stage of a swollen carpel (Fig 1C). Taken together, these data suggest that reversion increases fecundity on a per flower level, but also that shorter days, which were used to increase reversion levels, may decrease overall seed mass on a per plant basis, which is compensated for by the increased seed mass from reverting branches thus resulting in equal seed mass in the two light conditions.

### Natural variation in populations of *A*. *suecica* is correlated with differences in reversion rate and flowering time

The allopolyploid species *A*. *suecica* was derived from a single allopolyploidization event between 12,000 and 300,000 years ago [23]. Given its unique origin, the relatively short time this species had to diversify into separate populations, and the possibility that floral reversion might be a favorable adaptive trait leading to enhanced fitness (Fig 3), we asked how much variation the species *A*. *suecica* has accumulated since speciation. We previously reported subtle, yet statistically significant variation of several, potentially adaptive phenotypes, including floral reversion, in eleven populations of *A*. *suecica*, and speculated that variation might have arisen from genomic stress during the original allopolyploidization event [31]. Given the diversity of ecological niches, in which *A*. *suecica* is found, and the photoperiod-dependency of floral reversion, we asked to what degree floral reversion is dependent on environmental conditions, inherent genetic diversity, and developmental stage. We confirmed and expanded the previous observation that frequency of floral reversion is increased in increasingly shorter days (Fig 4). The observed variation in reversion rate and flowering time between populations (S3 and S4 Figs) suggested to us that each population either adapted to local conditions differently over the short evolutionary time since divergence from the single neoallopolyploid, or that variable genetic or genomic change that occurred during the allopolyploidization event itself was passed down to varying degrees to the different siblings in the first allopolyploid generation. The latter scenario is consistent with multiple recent studies reporting that hybridization and allopolyploidy coincide with major genomic and genetic alterations [32–34]. Although our data do not show this directly, it is possible that the original neoallopolyploid population already harbored some of the variation that later gave rise to the phenotypically variable populations of *A*. *suecica* that we see today. *A*. *suecica* populations are widely distributed across Scandinavia, where day lengths can vary substantially between locations in the summer during the plants’ bloom. To test the possibility that the observed differences in variation of reversion rates are due to a latitudinal cline of the sampling locations, we used Pearson analysis but found no significant correlation between reversion and latitude or between flowering time and latitude (data not shown). Several studies in the past have shown that flowering time in *A*. *thaliana* is correlated with latitude [35,36], however the latitudinal cline in our study was considerably smaller (59–65°) compared to that in similar studies in *A*. *thaliana* where flowering time increased with latitude from 40–65° [36–38].

### Day length effects the developmental position of floral reversions and flowering time in *A*. *suecica*

Our data show that occurrence of reverting flowers is non-random along the inflorescence axis and affected by day length (Fig 5). Likewise, we show that flowering time in *A*. *suecica* is day-length dependent (Fig 6) with a large difference between 8 and 12 hours, but only small differences between 12,16, and 24 hours of light, suggesting that a threshold between 8 and 12 hours of day length results in a disproportionally promoting effect on flowering. We asked to what degree floral reversion is dependent on environmental conditions. Given a higher overall number of reverting flowers in plants grown in short days (Fig 4) but also a later onset of flowering in plants exposed to shorter photoperiods we statistically tested the significance of this relationship (Fig 7). Indeed, reversion frequency is positively correlated with flowering time in *A*. *suecica*, suggesting that plants taking longer to pass the required threshold to flowering are more likely not to transition directly to producing terminal flowers along the inflorescence and instead spend additional time in transition, thus increasing the likelihood of reversion.

To further investigate the question of causality of reversion we hypothesized that differences in developmental age might be the reason why some flowers revert while others do not. Distribution of seed quantity and other fruiting or flowering traits among the flowers of an individual plant is usually not uniform and often decreases in magnitude or size from base to apex of a plant as a result of resource limitation, competition, or the inflorescence architecture itself [39,40]. Given that floral reversion is most frequent during the mid-phase of inflorescence development, it is unlikely that resource limitation could explain the phenomenon. Instead, we hypothesized that floral reversion is determined by developmental genes that are involved in the transition from inflorescence meristems (IM) to floral meristems (FM). The fact that the peak position of floral reversion is photoperiod-dependent (Fig 5) suggested to us that genes involved both in the photoperiod response and the transition from IM to FM might be mis-regulated in *A*. *suecica*, leading to the unstable state of floral reversion. Genes involved in floral transition and photoperiodism have been well-characterized in *A*. *thaliana* [41–44]. By tightly controlling the transition from IM to FM via an intricate feedback loop *AGAMOUS-LIKE 24* (*AGL24*) and *SHORT VEGETATIVE PHASE* (*SVP*) [45–47] ensure that terminal floral meristems are not produced too soon. Flower initiation in *A*. *arenonsa* occurs later than in *A*. *thaliana* (unpublished observations); it is therefore tempting to speculate that homoeologous proteins expressed from one genome (for example the earlier flowering *A*. *thaliana*) are ready to prepare the plant for the transition from IM to FM, while the homoeologs of the later flowering parental genome are still repressing this transition. Trans-regulation of one parental genome by transcription factors from the other parental genome in the allopolyploid hybrid could in this scenario explain the tension between delay and promotion of the transition to a FM, leading to mis-regulation of flower initiation genes and the transitory floral reversion phenotype. Several homoeologs of the flower regulating genes have been identified in *A*. *arenosa* and in some cases trans-activation of one genome by transcription factors of the other genome, and vice versa, has been reported [48]. It is therefore formally possible that such trans-activation might be the reason for floral reversion in *A*. *suecica*.

In conclusion, our results suggest that flower induction, floral reversion, and day length might be interconnected leading to the re-initiation of growth from the replum. Future and ongoing work on this phenomenon will further address the question of trans-regulation of homoeologs in the allopolyploid genome, and focus on transcriptomic differences that underlie floral reversion.

## Supporting Information

S1 Fig Growth temperature, but not day length, affects whole plant seed weight. Plants were grown in incubators either at 20°C or 25°C at the indicated day length conditions. Siliques were harvested as they were ripening and sorted into separate envelopes from each plant. Total seed weight was measured after plants had senesced. ANOVA and Tukey posthoc analysis showed that light treatment at constant temperature has no effect on seed weight (p> 0.05), while seed mass differs in plants grown at different temperatures (p< 0.05). Treatments not connected by the same letter are statistically significantly different from each other. (N = 23, 15, 21, 41). Error bars reflect SE.
(TIF)

S2 Fig Floral reversion frequency varies between populations and is increased in its frequency in short days. Individuals from each population were grown in incubators (20°C; and day lengths set at 8h light/16h dark; 12h light/12h dark; 16h light/8h dark; 24h light) until senescence. Reverting flowers were counted along the inflorescence axis from the first to the 50th flower and the proportion of reverting flowers calculated. Statistically significant differences in reversion rates were determined by ANOVA for each population. Generally, statistically significant differences were observed in some populations between the shortest and the longest light treatments only. The complete ANOVA results are reported in S2 Table. Populations are sorted left to right from the southern-most to the northern-most origin. Error bars reflect SE. N varied between time points and populations as follows: 8h: N = 2, 12h: N: = 10.3 (6–15), 16h: N = 9.7 (9–11), 24h: N = 4.
(TIF)

S3 Fig Flowering time is significantly increased in shorter days. Plants were grown in growth chambers at the indicated day lengths. The first day of flowering was defined as the day when the first flower opened or the inflorescence was 1 cm tall, whichever occurred first. ANOVAs were performed for each population (for complete ANOVA results see S3 Table). Populations are sorted left to right from the southern-most to northern-most origin. Error bars reflect SE. N varied between time points and populations as follows: 8h: N = 3.8 (2–4), 12h: N: = 7 (5–10), 16h: N = 7.8 (7–8), 24h: N = 5.8 (5–6).
(TIF)

S4 Fig Floral reversion is positively correlated with flowering time. This figure includes all data from Fig 7, as well as the data from plants on which no flower reverted, which was removed in Fig 7. Flowering time and reversion data were plotted for each individual plant, and the Pearson correlation coefficient was calculated (p< 0.0001, R^2^ = 0.1083, N = 190). Using this data set, the correlation was positive and statistically significant, yet less strong than when non-reverting plants were removed from the analysis as in Fig 7.
(TIF)

S1 Table Populations used in analysis of reversion rates and flowering time. (DOCX)

S2 Table ANOVA results for differences in reversion frequencies between light treatments in each of the experimental populations. (DOCX)

S3 Table ANOVA results for differences in flowering time between light treatments in the experimental populations. (DOCX)

S4 Table Raw data for all reversion and flowering time analyses. (XLSX)
