# Supplementary figures and images for: Floral Reversion in Arabidopsis suecica Is Correlated with the Onset of Flowering and Meristem Transitioning
Source: PLoS One. 2015 May 26;10(5):e0127897. doi: 10.1371/journal.pone.0127897 (PMC4444321; doi:10.1371/journal.pone.0127897)

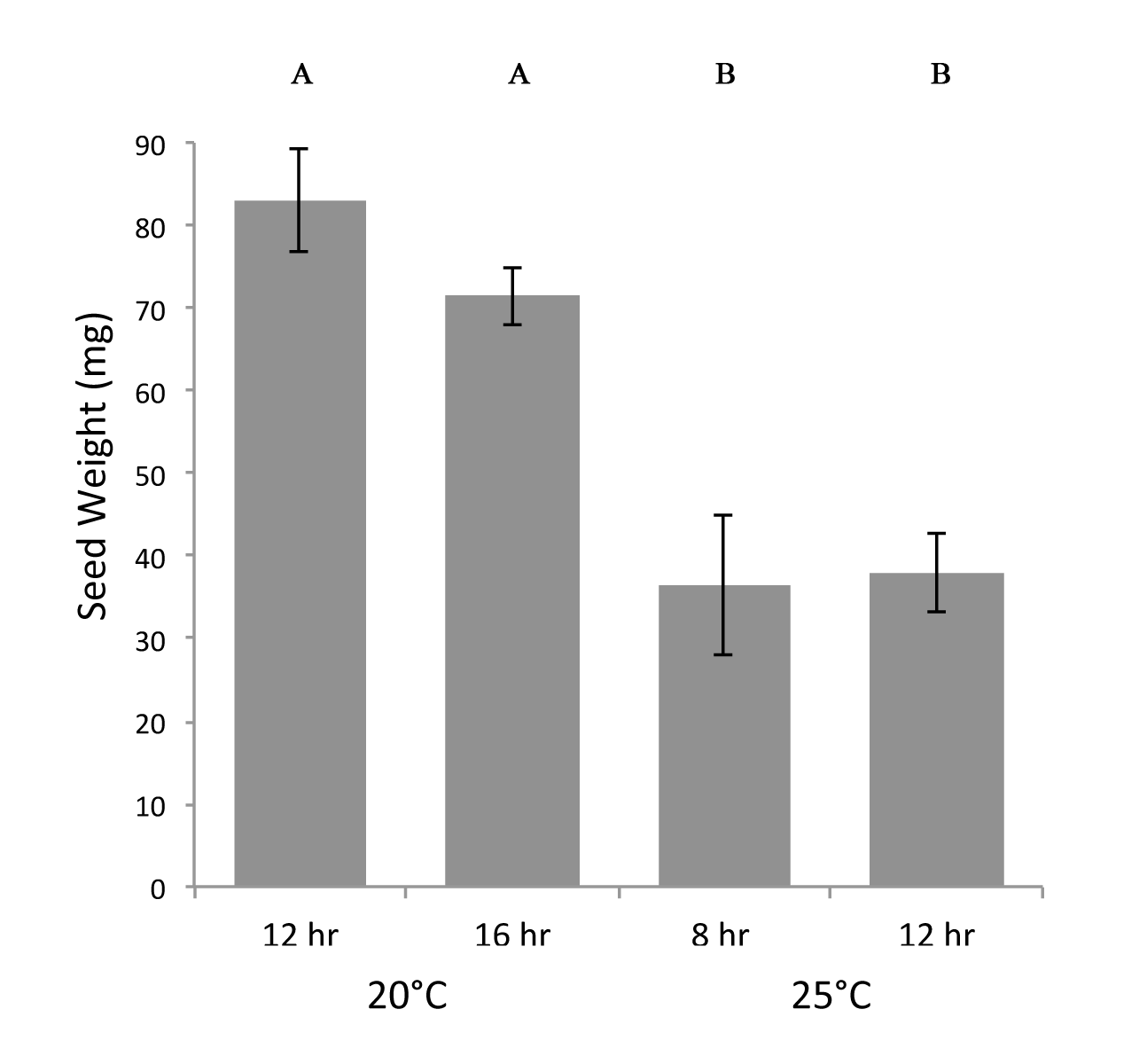

Supplement: S1 Fig — Plants were grown in incubators either at 20°C or 25°C at the indicated day length conditions. Siliques were harvested as they were ripening and sorted into separate envelopes from each plant. Total seed weight was measured after plants had senesced. ANOVA and Tukey posthoc analysis showed that light treatment at constant temperature has no effect on seed weight (p> 0.05), while seed mass differs in plants grown at different temperatures (p< 0.05). Treatments not connected by the same letter are statistically significantly different from each other. (N = 23, 15, 21, 41). Error bars reflect SE. (TIF) [file pone.0127897.s001.tif]

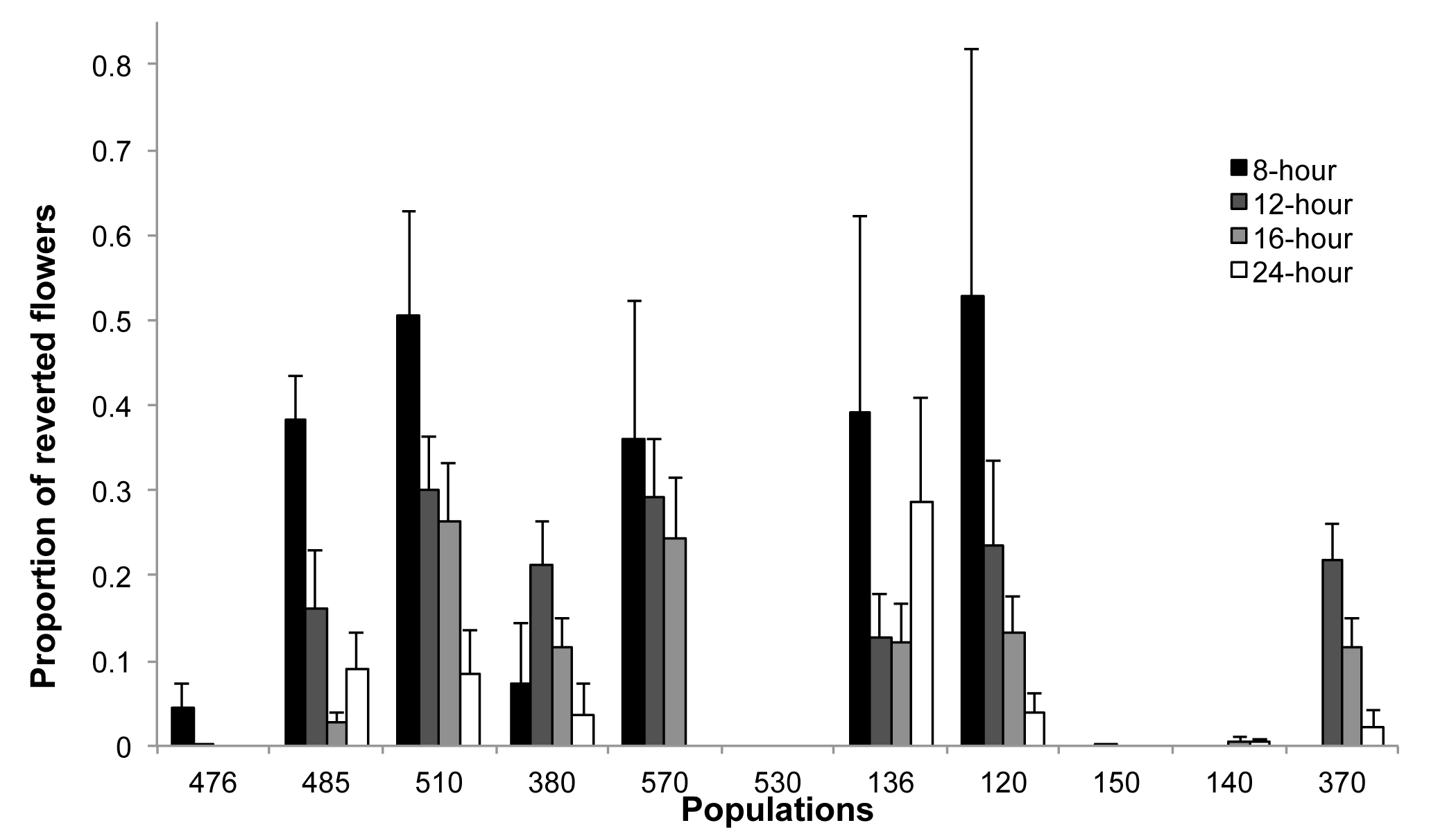

Supplement: S2 Fig — Individuals from each population were grown in incubators (20°C; and day lengths set at 8h light/16h dark; 12h light/12h dark; 16h light/8h dark; 24h light) until senescence. Reverting flowers were counted along the inflorescence axis from the first to the 50th flower and the proportion of reverting flowers calculated. Statistically significant differences in reversion rates were determined by ANOVA for each population. Generally, statistically significant differences were observed in some populations between the shortest and the longest light treatments only. The complete ANOVA results are reported in S2 Table. Populations are sorted left to right from the southern-most to the northern-most origin. Error bars reflect SE. N varied between time points and populations as follows: 8h: N = 2, 12h: N: = 10.3 (6–15), 16h: N = 9.7 (9–11), 24h: N = 4. (TIF) [file pone.0127897.s002.tif]

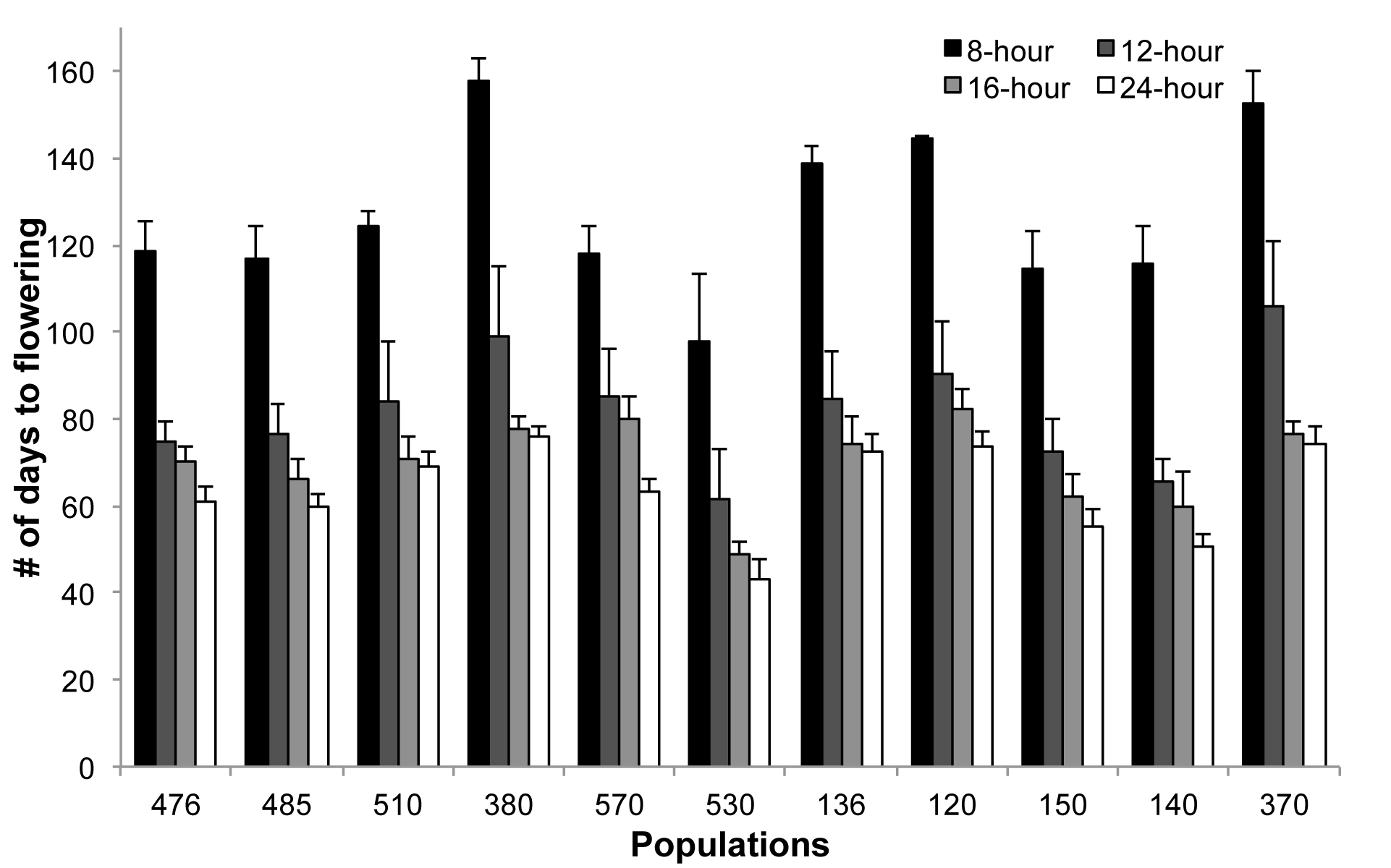

Supplement: S3 Fig — Plants were grown in growth chambers at the indicated day lengths. The first day of flowering was defined as the day when the first flower opened or the inflorescence was 1 cm tall, whichever occurred first. ANOVAs were performed for each population (for complete ANOVA results see S3 Table). Populations are sorted left to right from the southern-most to northern-most origin. Error bars reflect SE. N varied between time points and populations as follows: 8h: N = 3.8 (2–4), 12h: N: = 7 (5–10), 16h: N = 7.8 (7–8), 24h: N = 5.8 (5–6). (TIF) [file pone.0127897.s003.tif]

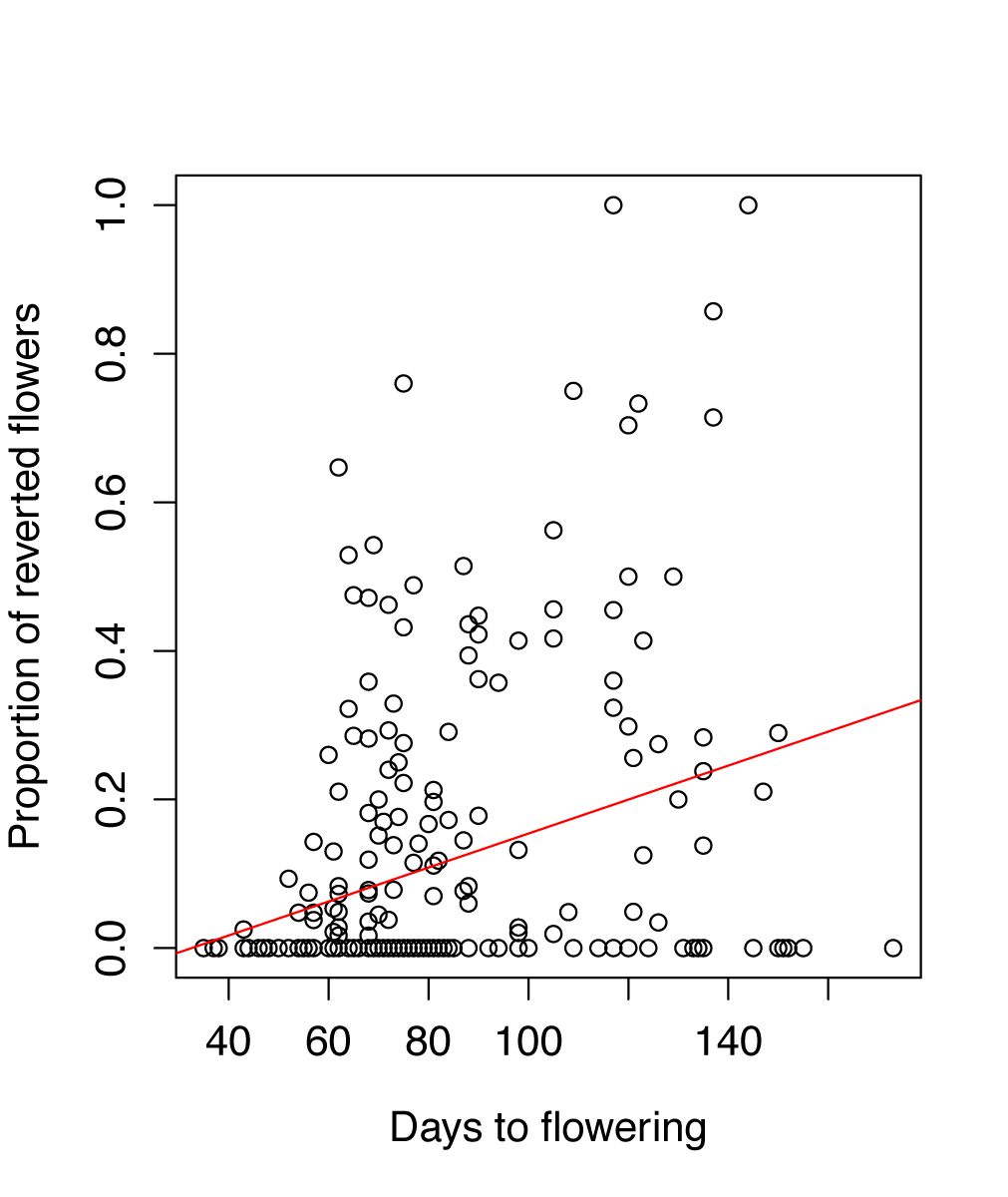

Supplement: S4 Fig — This figure includes all data from Fig 7, as well as the data from plants on which no flower reverted, which was removed in Fig 7. Flowering time and reversion data were plotted for each individual plant, and the Pearson correlation coefficient was calculated (p< 0.0001, R2 = 0.1083, N = 190). Using this data set, the correlation was positive and statistically significant, yet less strong than when non-reverting plants were removed from the analysis as in Fig 7. (TIF) [file pone.0127897.s004.tif]
