# Supplementary material for: Floral Reversion in Arabidopsis suecica Is Correlated with the Onset of Flowering and Meristem Transitioning
Source: PLoS One. 2015 May 26;10(5):e0127897. doi: 10.1371/journal.pone.0127897 (PMC4444321; doi:10.1371/journal.pone.0127897)
Supplement: S1 Table — (DOCX) [file pone.0127897.s005.docx]

S1 Table **Populations used in analysis of reversion rates and flowering time.**

| **Collection Number** | **Lat (N)** | **Long (E)** | **City/Town** |
| --- | --- | --- | --- |
| 476 | 59 51 48 | 17 49 04 | Bärby |
| 485 | 59 52 58 | 18 02 83 | Almunge |
| 510 | 60 11 | 24 59 | Helsinki |
| 380 | 60 12 | 25 50 | Helsinki |
| 570 | 60 46 | 16 57 | Oslättfors |
| 530 | 61 15 | 24 20 | Pälkäne |
| 136 | 61 52 54 | 17 19 17 | Strömsbruk |
| 120 | 61 53 68 | 16 32 51 | Friggesund |
| 150 | 62 10 37 | 14 56 03 | Ytterhogdal |
| 140 | 62 36 18 | 17 02 33 | V. Indal |
| 370 | 65 00 | 25 00 | Oulu |
