# Supplementary material for: Floral Reversion in Arabidopsis suecica Is Correlated with the Onset of Flowering and Meristem Transitioning
Source: PLoS One. 2015 May 26;10(5):e0127897. doi: 10.1371/journal.pone.0127897 (PMC4444321; doi:10.1371/journal.pone.0127897)
Supplement: S2 Table — (DOCX) [file pone.0127897.s006.docx]

S2 Table **Populations used in analysis of reversion rates and flowering time**

| **Population** | **Df** | **F value** | **p-value of F-test** | **Tukey comparison** | **Tukey HSD p-value** | **Sign at 0.05?** |
| --- | --- | --- | --- | --- | --- | --- |
| 485 | 3,23 | 4.231 | 0.0161 | 12h-8h | 0.0771877 | N |
| 485 |  |  |  | 16h-8h | 0.0084817 | Y |
| 485 |  |  |  | 24h-8h | 0.1963357 | N |
| 485 |  |  |  | 16h-12h | 0.3517972 | N |
| 485 |  |  |  | 24h-12h | 0.9999996 | N |
| 485 |  |  |  | 24h-16h | 0.5715407 | N |
|  |  |  |  |  |  |  |
| 510 | 3,21 | 1.836 | 0.172 | 12h-8h | 0.3987181 | N |
| 510 |  |  |  | 16h-8h | 0.6277506 | N |
| 510 |  |  |  | 24h-8h | 0.1316143 | N |
| 510 |  |  |  | 16h-12h | 0.9713762 | N |
| 510 |  |  |  | 24h-12h | 0.6210008 | N |
| 510 |  |  |  | 24h-16h | 0.4590133 | N |
|  |  |  |  |  |  |  |
| 380 | 3,13 | 0.893 | 0.471 | 12h-8h | 0.8805314 | N |
| 380 |  |  |  | 16h-8h | 0.9999986 | N |
| 380 |  |  |  | 24h-8h | 0.945699 | N |
| 380 |  |  |  | 16h-12h | 0.4422972 | N |
| 380 |  |  |  | 24h-12h | 0.9999998 | N |
| 380 |  |  |  | 24h-16h | 0.8795464 | N |
|  |  |  |  |  |  |  |
| 570 | 2,15 | 0.067 | 0.935 | 12h-8h | 0.9321377 | N |
| 570 |  |  |  | 16h-8h | 0.9787618 | N |
| 570 |  |  |  | 24h-8h | No 24h reversion |  |
| 570 |  |  |  | 16h-12h | 0.9771592 | N |
| 570 |  |  |  | 24h-12h | No 24h reversion |  |
| 570 |  |  |  | 24h-16h | No 24h reversion |  |
|  |  |  |  |  |  |  |
| 136 | 3,14 | 4.875 | 0.0159 | 12h-8h | 0.0325227 | Y |
| 136 |  |  |  | 16h-8h | 0.0209664 | Y |
| 136 |  |  |  | 24h-8h | 0.4014673 | N |
| 136 |  |  |  | 16h-12h | 0.9870171 | N |
| 136 |  |  |  | 24h-12h | 0.3128802 | N |
| 136 |  |  |  | 24h-16h | 0.2013955 | N |
|  |  |  |  |  |  |  |
| 120 | 3,12 | 3.603 | 0.046 | 12h-8h | 0.1166148 | N |
| 120 |  |  |  | 16h-8h | 0.0483802 | Y |
| 120 |  |  |  | 24h-8h | 0.0529345 | N |
| 120 |  |  |  | 16h-12h | 0.9401029 | N |
| 120 |  |  |  | 24h-12h | 0.7289967 | N |
| 120 |  |  |  | 24h-16h | 0.9050465 | N |
|  |  |  |  |  |  |  |
| 370 | 2,15 | 0.409 | 0.672 | 12h-8h | no 8h reversions | |
| 370 |  |  |  | 16h-8h | no 8h reversions | |
| 370 |  |  |  | 24h-8h | no 8h reversions | |
| 370 |  |  |  | 16h-12h | 0.8489752 | N |
| 370 |  |  |  | 24h-12h | 0.7072897 | N |
| 370 |  |  |  | 24h-16h | 0.8636432 | N |
