# Supplementary material for: Floral Reversion in Arabidopsis suecica Is Correlated with the Onset of Flowering and Meristem Transitioning
Source: PLoS One. 2015 May 26;10(5):e0127897. doi: 10.1371/journal.pone.0127897 (PMC4444321; doi:10.1371/journal.pone.0127897)
Supplement: S3 Table — (DOCX) [file pone.0127897.s007.docx]

S3 Table **ANOVA results for differences in flowering time between light treatments in the experimental populations.**

| **Population** | **Df** | **F value** | **p-value of F-test** | **Tukey comparison** | **Tukey HSD p-value** | **Sign at 0.05?** |
| --- | --- | --- | --- | --- | --- | --- |
| 476 | 3,24 | 16.14 | 5.90E-06 | 12h-8h | 0.0001229 | Y |
| 476 |  |  |  | 16h-8h | 0.0000398 | Y |
| 476 |  |  |  | 24h-8h | 0.0000037 | Y |
| 476 |  |  |  | 16h-12h | 0.8462895 | N |
| 476 |  |  |  | 24h-12h | 0.1161203 | N |
| 476 |  |  |  | 24h-16h | 0.4393593 | N |
|  |  |  |  |  |  |  |
| 485 | 3,22 | 23.62 | 4.52E-07 | 12h-8h | 0.0000126 | Y |
| 485 |  |  |  | 16h-8h | 0.0000016 | Y |
| 485 |  |  |  | 24h-8h | 0.0000005 | Y |
| 485 |  |  |  | 16h-12h | 0.4674359 | N |
| 485 |  |  |  | 24h-12h | 0.1164821 | N |
| 485 |  |  |  | 24h-16h | 0.8095474 | N |
|  |  |  |  |  |  |  |
|  |  |  |  |  |  |  |
| 510 | 3,19 | 13.48 | 5.99E-05 | 12h-8h | 0.0021112 | Y |
| 510 |  |  |  | 16h-8h | 0.0000654 | Y |
| 510 |  |  |  | 24h-8h | 0.0001375 | Y |
| 510 |  |  |  | 16h-12h | 0.4348871 | N |
| 510 |  |  |  | 24h-12h | 0.4267091 | N |
| 510 |  |  |  | 24h-16h | 0.9973758 | N |
|  |  |  |  |  |  |  |
| 380 | 3,19 | 21.88 | 2.10E-05 | 12h-8h | 0.0005651 | Y |
| 380 |  |  |  | 16h-8h | 0.0000028 | Y |
| 380 |  |  |  | 24h-8h | 0.0000045 | Y |
| 380 |  |  |  | 16h-12h | 0.1488763 | N |
| 380 |  |  |  | 24h-12h | 0.1455549 | N |
| 380 |  |  |  | 24h-16h | 0.9984553 | N |
|  |  |  |  |  |  |  |
| 570 | 3,20 | 3.7 | 0.0285 | 12h-8h | 0.6363451 | N |
| 570 |  |  |  | 16h-8h | 0.3100651 | N |
| 570 |  |  |  | 24h-8h | 0.0210552 | Y |
| 570 |  |  |  | 16h-12h | 0.933151 | N |
| 570 |  |  |  | 24h-12h | 0.1437385 | N |
| 570 |  |  |  | 24h-16h | 0.2962105 | N |
|  |  |  |  |  |  |  |
| 530 | 3,20 | 25.92 | 4.28E-07 | 12h-8h | 0.0000361 | Y |
| 530 |  |  |  | 16h-8h | 0.0000008 | Y |
| 530 |  |  |  | 24h-8h | 0.0000006 | Y |
| 530 |  |  |  | 16h-12h | 0.3497257 | N |
| 530 |  |  |  | 24h-12h | 0.1456471 | N |
| 530 |  |  |  | 24h-16h | 0.896374 | N |
|  |  |  |  |  |  |  |
| 136 | 3,19 | 20.32 | 3.76E-06 | 12h-8h | 0.0001392 | Y |
| 136 |  |  |  | 16h-8h | 0.000005 | Y |
| 136 |  |  |  | 24h-8h | 0.0000071 | Y |
| 136 |  |  |  | 16h-12h | 0.6554424 | N |
| 136 |  |  |  | 24h-12h | 0.5742925 | N |
| 136 |  |  |  | 24h-16h | 0.9956941 | N |
|  |  |  |  |  |  |  |
| 120 | 3,15 | 8.598 | 0.00147 | 12h-8h | 0.0153858 | Y |
| 120 |  |  |  | 16h-8h | 0.0028433 | Y |
| 120 |  |  |  | 24h-8h | 0.0009039 | Y |
| 120 |  |  |  | 16h-12h | 0.7690948 | N |
| 120 |  |  |  | 24h-12h | 0.261129 | N |
| 120 |  |  |  | 24h-16h | 0.6914061 | N |
|  |  |  |  |  |  |  |
| 150 | 3,24 | 14.72 | 2.00E-05 | 12h-8h | 0.000735 | Y |
| 150 |  |  |  | 16h-8h | 0.0000447 | Y |
| 150 |  |  |  | 24h-8h | 0.0000143 | Y |
| 150 |  |  |  | 16h-12h | 0.4927892 | N |
| 150 |  |  |  | 24h-12h | 0.1426191 | N |
| 150 |  |  |  | 24h-16h | 0.8087581 | N |
|  |  |  |  |  |  |  |
| 140 | 3,24 | 19.91 | 1.07E-06 | 12h-8h | 0.0000097 | Y |
| 140 |  |  |  | 16h-8h | 0.0000042 | Y |
| 140 |  |  |  | 24h-8h | 0.0000012 | Y |
| 140 |  |  |  | 16h-12h | 0.8883779 | N |
| 140 |  |  |  | 24h-12h | 0.3060629 | N |
| 140 |  |  |  | 24h-16h | 0.7093602 | N |
|  |  |  |  |  |  |  |
| 370 | 3,19 | 18.75 | 6.65E-06 | 12h-8h | 0.0029464 | Y |
| 370 |  |  |  | 16h-8h | 0.0000107 | Y |
| 370 |  |  |  | 24h-8h | 0.0000132 | Y |
| 370 |  |  |  | 16h-12h | 0.030916 | Y |
| 370 |  |  |  | 24h-12h | 0.0291211 | Y |
| 370 |  |  |  | 24h-16h | 0.9949103 | N |
